# A propos d’un hémangiome cutané compliqué traité en ambulatoire

**DOI:** 10.11604/pamj.2023.45.126.40615

**Published:** 2023-07-14

**Authors:** Hajar El Kouarty, Badr Sououd Benjelloun Dakhama

**Affiliations:** 1 Service des Urgences Médicales Pédiatriques, Hôpital d'Enfants Rabat, Université Mohammed V, Rabat, Maroc

**Keywords:** Hémangiomes, enfants, béta bloquant, Hemangioma, children, beta-blockers

## Abstract

Cutaneous hemangiomas are benign vascular tumors, they can be complicated by very hemorrhagic ulcers and rarely by infections. Oral treatment with beta-blockers has revolutionized the management of complicated cutaneous hemangiomas. We report a case of a 4-month-old infant with no notable history, who consulted for purulent ulceration of a cutaneous hemangioma in the upper of thorax with fever from 3 days ago. The clinical examination finds a febrile infant at 39°C, the hemangioma of 10 cm large presents a central necrosis (A). The indication of hospitalization for management was indicated, but refused by the parents. The biological assessment revealed a CRP at 124mg/l with hyperleukocytosis at 15700/mm^3^ predominantly PNN. We started the outpatient treatment: daily local care of the infected ulceration, oral antibiotic therapy: Amoxicillin + clavulanic acid for 10 days and analgesics (Paracetamol). Faced with the hemorrhagic appearance of the ulceration measuring 3cm large diameter (B), we started the beta-blockers, outpatient, orally, after performing a normal ECG. (1mg/kg/day then 2mg/kg/day in two doses after the 1^st^ week then 3mg/kg/day the third week). Twice-daily monitoring of capillary blood glucose, blood pressure and heart rate was performed at each dose increase. Clinical improvement was spectacular with significant involution of hemangioma (C) after 2 months of treatment with Propranolol. This observation recalls the importance of the introduction of beta-blockers in front of complicated hemangiomas as well as the possibility of their management on an outpatient basis.

## Image en médecine

Les hémangiomes cutanés sont des tumeurs vasculaires bénignes, ils peuvent se compliquer d´ulcères très hémorragiques et rarement d´infection. Le traitement oral par bétabloquants a révolutionné la prise en charge des hémangiomes cutanés compliqués. Nous rapportons le cas d´un nourrisson de 4 mois sans antécédents notables, ayant consulté pour ulcération d´un hémangiome cutané de la partie supérieur du thorax avec issu de pus et fièvre depuis 3 jours. L´examen clinique retrouve un nourrisson fébrile à 39°C, l´hémangiome de 10cm de grand diamètre présente une nécrose centrale (A). L´indication de l´hospitalisation pour prise en charge a été indiquée, mais refusée par les parents. Le bilan a révélé une CRP à 124mg/l avec hyperleucocytose à 15700/mm^3^ à prédominance PNN. Nous avons donc démarré le traitement en ambulatoire: soins locaux quotidiens de l´ulcération infectée, antibiothérapie par voie orale: amoxicilline + acide clavulanique pendant 10 jours et antalgiques (paracétamol). Devant l´aspect hémorragique de l´ulcération mesurant 3cm de grand diamètre (B), nous avons démarré les bétabloquants, en ambulatoire, par voie orale, après réalisation d´un ECG normal. (1mg/kg/j puis 2mg/kg/j en deux prises après la 1^ère^ semaine puis 3mg/kg/j la troisième semaine). La surveillance biquotidienne de la glycémie capillaire, de la tension artérielle et de la fréquence cardiaque, était réalisée à chaque augmentation de dose. L´amélioration clinique était spectaculaire avec involution importante de l´hémangiome (C) après 2 mois de traitement par Propranolol. Cette observation rappelle l´importance de l´introduction des bétabloquants devant des hémangiomes compliqués ainsi que la possibilité de leur prise en charge en ambulatoire.

**Figure 1:**
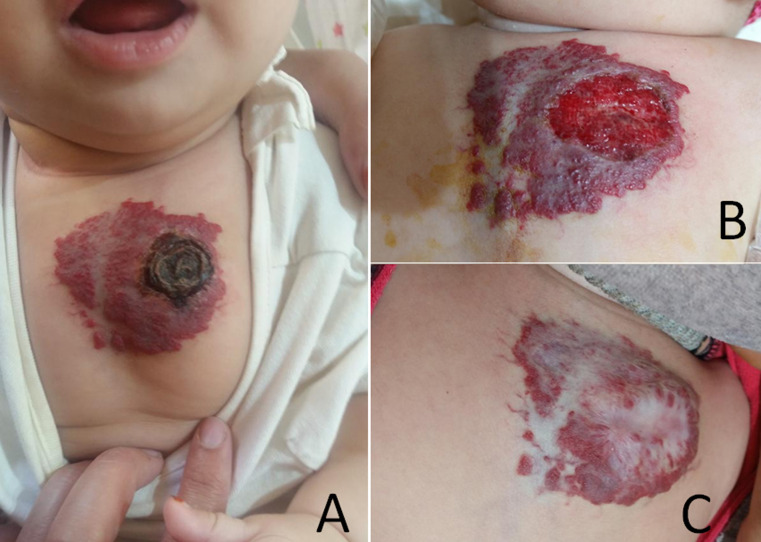
A,B,C) hémangiome ulcéré nécrosé avant et après traitement

